# A qualitative study of the impact of the COVID-19 pandemic on healthcare access and service delivery for people living with chronic obstructive pulmonary disease and asthma

**DOI:** 10.1186/s13223-026-01052-y

**Published:** 2026-06-26

**Authors:** Katelyn Brehon, Mirsol Choi, Pam Hung, Maxi Miciak, Rachel K. Lim, Mohit Bhutani, Kadija Perreault, Paul E. Ronksley, Micheal K. Stickland, Jason Weatherald, Douglas P. Gross, Grace Y. Lam

**Affiliations:** 1 Faculty of Rehabilitation Medicine, University of Alberta, Edmonton, AB T6G 2G4 Canada; 2 Department of Medicine, Faculty of Medicine and Dentistry, University of Alberta and Alberta Health Services, Edmonton, AB T6G 2G3 Canada; 3 School of Rehabilitation Science, University of Saskatchewan, Saskatoon, SK S7N 5A2 Canada; 4 Division of Respiratory Medicine, Department of Medicine, Cumming School of Medicine, University of Calgary and Alberta Health Services, Calgary, AB T2N 1N4 Canada; 5 École des sciences de la réadaptation, Université Laval, Québec, QC G1V 0A6 Canada; 6 Centre for Interdisciplinary Research in Rehabilitation and Social Integration, Centre intégré universitaire de santé et de services sociaux de la Capitale-Nationale, Québec, QC G1M 2S8 Canada; 7 Department of Community Health Sciences, Cumming School of Medicine, University of Calgary, Calgary, AB T2N 4Z6 Canada; 8 O’Brien Institute for Public Health, University of Calgary, Calgary, AB T2N 4Z6 Canada; 9 Department of Physical Therapy, University of Alberta, Edmonton, AB T6G 2G4 Canada

**Keywords:** COPD, Asthma, Qualitative, Access, Quality, Interpretive

## Abstract

**Background:**

Incidence of chronic obstructive pulmonary disease and asthma diagnosis were lower during and after the Coronavirus disease 2019 pandemic in Alberta, Canada. However, it is unknown whether incidences were actually lower or if the pandemic created circumstances where patients did not seek care. As such, the objective of the current study was to explore the impact of COVID-19 on patient and clinician experiences of healthcare access and delivery.

**Methods:**

The study was conducted between October 2023 and July 2024. We used interpretive description, a qualitative approach with the end-goal of informing clinical decisions. Analysis was informed by Braun and Clarke’s six phases of reflexive thematic analysis.

**Results:**

We completed thirteen interviews. Two key themes were generated: (1) The pandemic impacted care-seeking behaviours; and (2) A time and place for virtual and in-person care. Clinicians discussed how access to entry points to the health system were impacted by the pandemic and highlighted how strategies to manage health and stressors impacted symptoms and subsequent care-seeking behaviours. Participants highlighted the positives of virtual and in-person care with the consensus that both are valuable.

**Conclusion:**

Future use of virtual care modalities should include a visual element at minimum and prioritize the therapeutic relationship.

## Background

Chronic obstructive pulmonary disease (COPD) and asthma are common pulmonary conditions resulting in small airways inflammation afflicting populations worldwide, with estimated prevalence of 10% and 5% respectively [1]. Management is often shared between family physicians and respirologists. Chronic disease management is essential to improving outcomes of patients and reducing risk of future acute exacerbations [2].

Prior research investigated how the COVID-19 pandemic impacted rates of COPD and asthma in Alberta, Canada. In a retrospective population-based analysis comparing 18 months prior to and following March 2020, Lam et al. found reduced incidence of COPD which the authors hypothesized may have been due to decreased access to in-person pulmonary function testing in order to mitigate COVID-19 exposure risk [3]. Similarly, after consistency for several years, the Canadian Chronic Disease Surveillance System reported a reduced incidence of asthma in Alberta between 2019 and 2020 and 2020–2021 (480 to 333 per 100,000, respectively) [4]. In Alberta’s publicly funded healthcare system, patients typically access care through a family physician (general practitioner), with referral to specialists such as respirologists when needed through the family physician. It remains unclear whether these observed declines reflect a true reduction in disease occurrence or reduced care-seeking and diagnostic activity during the pandemic.

We were unable to find qualitative studies investigating the impact of the COVID-19 pandemic on healthcare access and care delivery for COPD or asthma patients. A better understanding of how the pandemic impacted care in these populations can shape future care provision. Qualitative research would help clarify the lived experiences of patients and clinicians during the pandemic to add additional context and support to previous and emerging quantitative work. Therefore, the objective of our study was to explore how the COVID-19 pandemic from 2020 to 2023 impacted patient and clinician experiences of healthcare access and delivery in Alberta, Canada for COPD and asthma.

## Materials and methods

This study was conducted in Alberta, Canada. Qualitative data was collected between October 2023 and July 2024. Ethics approval was obtained from the University of Alberta’s Health Research Ethics Board (Pro00118367) and the University of Calgary’s Conjoint Health Research Ethics Board (pSite-22-0025). All participants provided informed verbal consent. This was part of a larger study with methods published elsewhere [5, 6].

### Researchers’ positionality

The study team was interdisciplinary and composed of clinicians (physician, occupational therapist, physical therapist) working in specialty respiratory clinics researchers and researchers (rehabilitation, public health, medicine) with diverse experience and professional backgrounds. A team member with a public health research background completed the interviews (KB). However, the interdisciplinary nature of the research team promoted rich analytic discussions between team members with diverse backgrounds that are familiar with clinical settings, helping to ensure interpretation was appropriate and relevant to the interdisciplinary clinical context of respiratory specialty care [7, 8].

### Methodological approach

We used interpretive description, a practical and flexible qualitative methodology developed to generate clinically meaningful insights from patient and clinician experiences [7–9]. Grounded in constructivism, interpretive description guided our choice to recruit both patients and clinicians to capture multiple perspectives relevant to care delivery, informed purposive sampling strategies to promote diversity in condition (COPD and asthma) and care role (physicians and allied health), and oriented analysis toward generating practical insights about health care access, service delivery, and care effectiveness. In interpretive description, findings are a co-construction of the researchers’ inductive interpretations of participant experiences within the context and knowledge of the applied discipline and the researchers’ positionality [7–9].

### Study population and recruitment

Common in interpretive description, purposive sampling directed participant recruitment to promote diversity in perspectives from both clinicians and patients that could speak directly to the phenomenon of interest [7] and contribute findings relevant to clinical practice. Potential participants were identified by MB and RKL through their COPD and asthma clinics in Alberta, Canada. Prospective participants were contacted by KB by telephone to provide study information and obtain informed consent. All participants were adult residents of Alberta, willing to participate in research, and able to provide informed consent. We recruited individuals who reported a diagnosis of COPD or asthma (patients), and clinicians who were providing care in COPD or asthma clinics prior to the COVID-19 pandemic, to ensure participants could speak to changes associated with the onset of the pandemic. Clinicians included physicians and members of the allied health team and were not required to have a specific level of experience or professional designation. English proficiency was required to participate in interviews. All participants received a nominal gift card as compensation for their time.

### Data generation

One-on-one semi-structured interviews, ranging in length from 23 to 59 min, were completed via videoconference (Zoom Version 6.1.11) or phone. Patients and clinicians were interviewed by KB with different interview guides addressing the topics of need for care, access to care, and quality and effectiveness of care, derived from Levesque et al. “Conceptual framework of access to health care,” [10] which outlines how people gain entry to and use health services while considering both health system factors (e.g., approachability, acceptability, availability, affordability, appropriateness) and patient factors (e.g. ability to perceive, seek, reach, pay, engage in care). Different interview guides were used for clinicians and patients to capture similarities and differences in perspectives and experiences, and to identify clinically relevant findings from each group, recognizing that experiences are diverse [7]. Data generation and analysis occurred concurrently, enabling determination of sample adequacy and iterative refinement to the interview guides, as needed [7]. Interviews were recorded, transcribed verbatim, reviewed for accuracy, and imported into NVivo Version 14 for analysis [11].

### Data analysis

Analysis was conducted using Braun and Clarke’s reflexive thematic analysis [12–15] within the interpretive description framework. This approach was chosen as interpretive description and reflexive thematic analysis are flexible and can be inductively-driven, while considering researcher subjectivity as an analytic resource that promotes transparency throughout the research process [16]. Following transcript cleaning and data familiarization, initial analytic thoughts and codes were developed inductively for each transcript, aligning with both interpretive description and reflexive thematic analysis (KB) [16]. Codes were reviewed and grouped by KB based on common patterns in the data within and between the clinician and patient transcripts. Themes and subthemes were named, defined, and exemplary quotes from each participant group were identified. KB discussed and refined the developing themes, sub-themes, and quotes with PH and the study team to support contextual relevance within clinical practice. Differences in interpretation of the themes and sub-themes were discussed and refined to support application of the findings to clinical practice. KB contacted each participant to solicit feedback on proposed themes to further support co-construction of the findings [16, 17]. Two patient participants agreed and provided feedback that aligned with the findings. However, we were not able to contact the clinician participants. Willing participants were asked about resonance of findings [17]. Questions or concerns were noted and reflected upon. Subsequent changes were made as necessary. Participant quotations were edited for readability, including the use of ellipses to emphasize central ideas while maintaining brevity and clarity.

### Rigour

Choosing interpretive description as our methodology promoted epistemological integrity. Interviewing both patients and clinicians as well as having participants reflect on how the findings resonated with their experiences enhanced representative credibility. Verbatim participant accounts contributed to thick description and analytic logic. Continual reflection through reflexive memoing and team discussions amongst the interdisciplinary group of clinicians and researchers during the research process enhanced interpretive authority. Discussions between KB and PH during analysis to refine the themes and sub-themes and discuss relevance to clinical practice further supported credibility. These facets promote trustworthiness and rigour [7].

## Results

### Participant characteristics

We completed ten patient interviews and three clinician interviews. Two clinicians provided care for both COPD and asthma patients and were able to comment on both conditions under the context of COVID-19. The third clinician provided asthma care only. Of the patient participants, 4 had COPD and 6 had asthma. Most patients with asthma (83.3%) self-identified as female whereas most COPD patients self-identified as male (75.0%). On average, COPD patient participants were 73.8 years old and asthma patient participants were 59.7 years old. Nine patient participants resided in urban areas and one in rural. fter ten patient interviews and three clinician interviews, the sample was deemed adequate for analysis as participants shared a range of experiences about care access during the pandemic.

### Impact of the pandemic on healthcare access, service delivery, and health

**Fig. 1:**
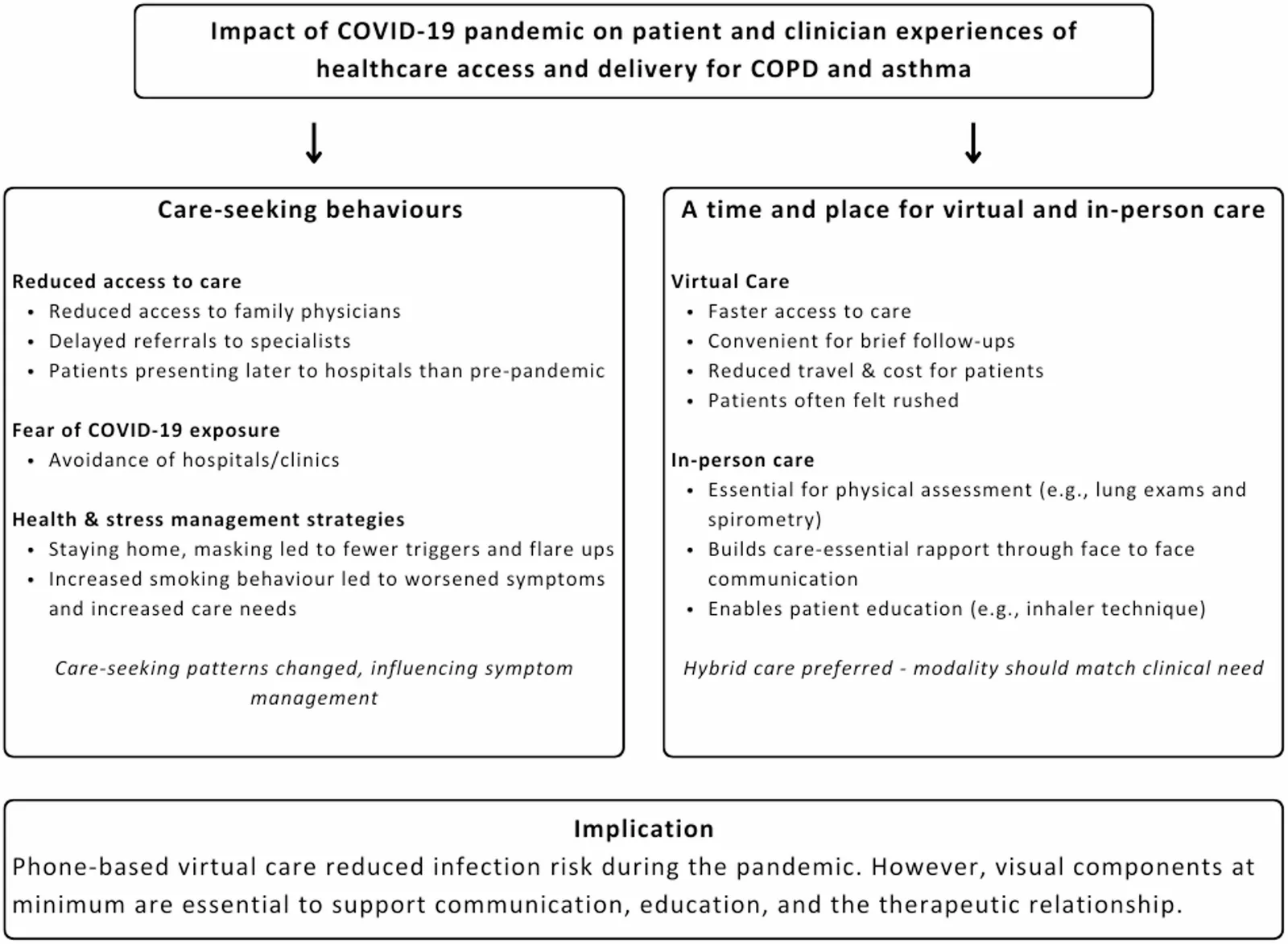
Two thematic outcomes from patient and clinician experiences in COPD and asthma care during the COVID-19 pandemic. COVID-19 Coronavirus disease 19; COPD Chronic obstructive pulmonary disease. Two thematic outcomes from the qualitative analysis of ten patient and three clinician interviews

The impact of the pandemic on healthcare access, service delivery, and patient health was explored through patients’ ability to access and receive COPD- or asthma-specific care, clinicians’ capacity to meet patient needs, and the overall symptoms and well-being reported by patient participants. Following thirteen interviews, two key themes emerged (Fig. 1).

#### The pandemic impacted care-seeking behaviours

The first theme describes how the pandemic impacted patients’ care-seeking behaviours.

##### Delays in accessing entry points to the healthcare system

Access to entry points into the healthcare system seemed to be more challenging for newly diagnosed COPD and asthma patients during the pandemic. Clinician participants described how some patients experienced challenges accessing family physicians, which created issues for any patient being managed exclusively by family physicians as referrals were often delayed to specialty care. This also created challenges for patients with more severe respiratory symptoms who are often co-managed by family physicians and a respirologist: *“I think everything slowed down for about the year*,* … you couldn’t access anybody because everything was closed pretty much. … So then the lines got longer.” (Asthma Clinician 1)*.

Patients and clinicians also described how individuals already diagnosed and connected with specialty care seemed to present later during the pandemic when struggling with symptoms compared to when they would have sought care prior to the pandemic. This delay was often attributed to fears of accessing healthcare and risking exposure to COVID-19: *“I think times where I probably would have went in sooner … we put it off … [I was] scared of going into the hospital with COVID. I was so scared I was going to get it on top of everything else.” (Asthma Patient 4)*.

If a patient sought care in-person during the pandemic, they described generally feeling safe in terms of exposures. Even if they did not want in-person care, patients generally felt confident about contacting their clinic for advice or recommendations: *“The door is always open to contact his office if I needed to” (Asthma Patient 6).* Patient participants believed they could receive care if needed: *“I’m sure that if I called them*,* that they would say*,* ‘Yeah*,* okay*,* we’ll book an appointment for you. Come on in’ and you know*,* I’m sure that’s the way it would be.” (COPD Patient 3)*.

##### Strategies to manage health and stressors during COVID-19 impacted symptom flare ups and care seeking

COPD and asthma patients and clinicians discussed how some patients’ strategies to manage their health and stressors (e.g., staying home more, wearing a mask) during the pandemic reduced symptom flare ups that reduced care-seeking. Others had strategies (i.e., smoking) that seemed to result in more flare ups, requiring more care during the pandemic. Those who experienced less symptom flare ups often attributed their better disease state to being exposed to less triggers during the pandemic: *“I didn’t actually have any acute episodes at that point. I wasn’t going out so I wasn’t … as exposed as some people” (Asthma Patient 1).*

These individuals were healthier overall and required less frequent access to healthcare services. In comparison, clinician participants described how some patients, especially in COPD, reverted to managing pandemic stress through unhealthy, triggering behaviours (i.e., smoking), thus impacting overall flare ups and need for care: *“maybe more so in COPD … when we talk about stressors … [they] can influence people’s ability to stay smoke–free … people with COPD … may … go back to taking up smoking*,* or smoke more if they’re already smoking*,* that can also happen with people with asthma as well. … so we did have some people … their lung symptoms got better. They were not exposed to … irritants*,* … viruses*,* or other respiratory infections … [so] their lungs were more stable. The others … maybe with … the stressors … may … have actually taken up … more smoking” (COPD/Asthma Clinician 1)*.

#### A time and place for virtual and in-person care

The second theme highlighted the benefits and drawbacks of virtual care, which was predominantly delivered over the telephone. Patient participants generally agreed they were thankful to be able to access any care during the pandemic, irrespective of modality (i.e., over the phone or in-person).

##### Virtual care is quicker and more convenient

COPD and asthma patients and clinicians discussed how virtual care was quicker and more convenient. Clinician participants in particular felt they were more willing to pick up the phone and call patients to check-in and/or communicate results during the pandemic thus providing quicker access: *“knowing that there is more comfort in phone … both [for] myself as well as the patient*,* … I may have a phone review sooner than I would’ve … [scheduled] an in-person visit” (COPD/asthma Clinician 1). “Nowadays that … we do a mix [of modalities]*,* I think it’s good*,* because some follow-up I think can be easily done over the phone. Sometimes you just want to see if somebody’s better. … some quick visits*,* especially for people that are from far away*,* I think the phone can be very useful.” (Asthma Clinician 1)*.

For patients living rurally or required to travel to attend specialist appointments, virtual care was valued for its ability to reduce travel costs and save time off work: *“We have a lot of people that come from far away. And they have to take two days off to come and see us. And in the past*,* … sometimes … it was like a 10-15-minute discussion*,* “What’s going on?”*,* they had to come all the way down here. And that’s cost for them.” (Asthma Clinician 1) “Well*,* … when you look at it and say*,* ‘Okay*,* I got a half hour drive to go to this … hospital’. And you know*,* I’m going to go see him and he’s going to say*,* ‘Oh*,* well*,* okay*,* everything is fine. Looks like we can just carry on for one more year.’ … for that*,* I’m fine with … talking to him on the phone.” (COPD Patient 3)*.

Some patients mentioned virtual appointments felt more rushed compared to in-person appointments, supporting the idea that convenience might not always be optimal: *“I feel like [with] the phone … it’s more like let’s have a brief conversation then get off the phone. And as much as it might seem more expedient*,* … sometimes you’re like I kind of need that time*” *(Asthma Patient 1).*

##### Effective assessments hinge on in-person elements

Patients and clinicians described the necessity of assessment still occurring in-person. Clinicians described how assessments over virtual care were only as good as the patient’s ability to report their symptoms, which varied significantly. Consensus was that patients valued clinicians being able to listen to their lungs and monitor them with regular testing: *“For COPD and asthma*,* spirometry and lung function testing [are] a very integral part of the assessment*,* and so … the clinical assessment was limited for many months in the early part of the pandemic. … there was only the ability to do … phone calls. And again*,* assessment is limited based on patients’ … symptom descriptions. No physical exam was possible*,* which … limited the completeness of the assessment.” (COPD/Asthma Clinician 1) “I thank heavens for the phone appointments if you can’t have anything else. But the in-person physical appointments where … they can have a look at you*,* where they can measure*,* … your breath and your intake as best as they can*,* and also listen to your lungs … I really did miss the in-person.” (Asthma Patient 4)*.

##### Visual elements of care are essential for quality communication and education

Patients valued communicating with clinicians face-to-face: *“It’s always nicer to speak to somebody face-to-face*,* or eye-to-eye” (COPD Patient 1)*. While phone was the main form of virtual connection during the pandemic, both patients and clinicians felt like phone conversations lacked emotional connection and rapport which made communication more challenging: *“I think I would’ve preferred Zoom*,* personally. … I think it’s something about being on the phone that … takes away from the human element” (Asthma Patient 3). “I guess you get more out of a patient when you have the ability to sit in front of them. You gain … a bit more rapport with them. You can kind of see … their mannerisms or things like that you pick up on when you have somebody in front of you.” (COPD/Asthma Clinician 2)*.

Clinicians in the study discussed how an essential component of COPD and asthma management is disease management education, specifically around inhaler techniques, smoking cessation, and breathing exercises to manage flare ups. Clinicians felt that a significant challenge with virtual care appointments during the pandemic was the limited visual component, which limited the ability to demonstrate items such as proper inhaler techniques thus impacting treatment effectiveness: *“And things like watching inhaler techniques*,* that was certainly not possible by phone*,* and so I’m reliant on either a description*,* or trying to send them a website video with inhaler techniques. But whether or not they were actually doing inhaler techniques correctly was unknown because of the limitations with virtual visits.” (COPD/Asthma Clinician 1)*.

## Discussion

We described COPD and asthma patients and clinician perceptions on access to, and effectiveness of, care delivered during the pandemic in Alberta, Canada. Both groups perceived delays in accessing the health system during the pandemic due to reduced access to family physicians and fears associated with COVID-19 exposure risk. While alternative care modalities were utilized during the pandemic and viewed as timelier and more convenient, patients sometimes felt rushed. Patients and clinicians valued in-person assessment as well as the relational and educational facets that come with having a visual element to providing care. Our findings suggest that previously observed reductions in COPD and asthma incidence likely reflect delays in care delivery, hesitancy in care-seeking, and limitations in physical assessment, rather than a true decline in disease occurrence.

Patients’ reduced access to family physicians and presenting later to care was not unique to our context and has been reported elsewhere [18–20]. Beyond reduced access, our patient participants chose to access care later than prior to the pandemic due to fear of contracting COVID-19. Other authors have reported asthma [21, 22] and COPD [23] patients delaying care during the pandemic due to perceived vulnerability to COVID-19.

Some patient participants, especially those with asthma, reported feeling the healthiest they had ever felt during the pandemic. Reduced asthma exacerbations during COVID-19 seems to be well established in the literature [21, 24–26]. In a retrospective cohort study investigating the impact of the pandemic on asthma in England, Shah and colleagues found exacerbation rates were consistently lower during the pandemic than before irrespective of sex, age, and region [26]. The authors attributed this reduction to public health measures and a reduction in circulating viruses [26].

Other participants noted that some patients’ stress management techniques were negatively impacted by the pandemic, especially among COPD patients. Similarly, in a cross-sectional study aimed at comparing the prevalence of depression, perceived stress related to COVID-19, post-traumatic stress, and insomnia among asthma and COPD patients in Columbia, the authors reported a similar frequency of the aforementioned challenges among asthma and COPD patients during lockdown [25]. These findings are especially important in terms of smoking and its relation to COPD. In a literature review about non-pharmacological treatments for COPD patients before and after the pandemic, Alqahtani et al. found that COVID-19-related stress increased the number of current smokers and made quitting more difficult [27].

Our participants discussed the convenience that virtual care offered during the pandemic but noted sometimes feeling rushed. Santillo and colleagues also reported that asthma patients with well-controlled symptoms appreciated telephone appointments for their flexibility and convenience [22]. However, Pinnock and colleagues noted that virtual care telephone consultations are generally shorter as they are often only addressing one problem and physicians have been found to devote less time to relationship building and providing education over the phone [28]. As such, the choice of whether COPD and asthma care should be delivered remotely or in-person in the post-pandemic context is complicated. Our participants overwhelmingly valued in-person care when assessments were needed. They also appreciated relational and educational aspects of care that came from some form of visual. Virtual care seemed to mainly be provided over the phone in the clinics recruited from so we do not know if the experience may be different if a video conferencing platform was used as part of virtual care delivery. Similar to clinician participants in our study, other authors [22, 25, 28] have noted that some face-to-face care is necessary to assess and for educational components to care, such as proper inhaler techniques.

## Limitations

Some limitations may limit the transferability of our results. First, we had a limited number of clinician participants due to recruitment challenges and health system pressures due to the ongoing pandemic at the time of data collection. However, two of the clinician participants provided both COPD and asthma care and therefore were able to comment on both conditions. Multiple attempts were made to recruit more clinician and patient participants but lack of willingness to participate and other feasibility issues prevented us from ongoing participant recruitment. Second, we also interviewed mainly patient participants who identified as male in the COPD cohort and female in the asthma cohort. However, more male participation for COPD and female participation for asthma is representative of the epidemiology of both diseases in our context. Concurrent analysis allowed us to determine sample adequacy based on the data from both clinicians and patient participants, recognizing that within interpretive description, we are not able to capture all possible variations of experiences [16]. Third, interviews were conducted remotely, meaning individuals without access to technology were inherently underrepresented. This could introduce bias, as participants with remote access may have experienced healthcare differently. Fourth, participation bias is possible, as individuals with particularly strong positive or negative experiences may have been more likely to participate. Fifth, interview length varied (23 to 59 min); however, all interviews remained conceptually rich and sufficient to meet the analytic aims. Finally, the temporal gap between pandemic experiences and data collection may have introduced recall bias. Despite these limitations, our study provides valuable insights into patient and clinician perspectives, and our measures such as purposive sampling, member reflections, and rigorous thematic analysis strengthens our findings.

## Conclusion

We investigated the impact of the COVID-19 pandemic on COPD and asthma healthcare access and care delivery. Entry points into the health system in both conditions were impacted by COVID-19. Alternative care modalities offered solutions to combat infection risk during pandemics, but virtual care cannot supplant in-person completely. Despite infrastructure and equity challenges limiting standardized implementation, future use of virtual care should strive to implement a visual component where appropriate and be tailored to the clinical context and patient preference, including new diagnoses, exacerbations, and patient education. In-person care should be prioritized when clinically necessary.

## Data Availability

The dataset generated and analysed during the current study is not publicly available as participants did not consent to sharing data publicly. The dataset can be made available from the corresponding author on reasonable request.
